# Optimization of The Electroporation Conditions for Transfection of Human Factor IX into The Goat Fetal Fibroblasts

**Published:** 2013-02-20

**Authors:** Amir Amiri Yekta, Azam Dalman, Mohammad Hossein Sanati, Nayeralsadat Fatemi, Hamed Vazirinasab, Alireza Zomorodipour, Mohammad Chehrazi, Hamid Gourabi

**Affiliations:** 1. Department of Genetics at Reproductive Biomedicine Research Center, Royan Institute for Reproductive Biomedicine, ACECR, Tehran, Iran; 2. Department of Embryology at Reproductive Biomedicine Research Center, Royan Institute for Reproductive Biomedicine, ACECR, Tehran, Iran; 3. Department of Medical Genetics, National Institute of Genetic Engineering and Biotechnology, Tehran, Iran; 4. Department of Molecular Genetics, National Institute of Genetic Engineering and Biotechnology, Tehran, Iran; 5. Department of Epidemiology and Reproductive Health at Reproductive Epidemiology Research Center, Royan Institute for Reproductive Biomedicine, ACECR, Tehran, Iran

**Keywords:** Gene Transfer, Naked DNA, Electroporation, Transgenic Animals, Fibroblast

## Abstract

**Objective:**

Electroporation is the most common method used for the transfection of DNA. Although this method has been attributed for various cell using different buffer compositions, the effects of DNA concentration on the transfection efficiency has not yet been studied. Here the correlation between the efficiency of electroporation reaction and increments of DNA concentration has been investigated. Following this investigation, a study was set out to produce a transgenic goat which is capable of secreting recombinant human coagulation factor IX in its milk.

**Materials and Methods:**

In this experimental study, a linearized recombinant vector (pBC1) entailing human coagulation factor IX (5.7 kb) cDNA was introduced into goat fetal fibroblast cells (three sub passages) via electroporation in four separate experiments (while other variables were kept constant), beginning with 10 µg DNA per pulse in the first experiment and increments of 10 µg/pulse for the next three reactions. Thereafter, polymerase chain reaction (PCR)-positive cell culture plates were diluted by several factors for further analysis of the transfected wells. Subsequently, positive cells were isolated for fluorescence *in situ* hybridization. Logistic regression model was used for data analyzing. Significance was defined as p< 0.05.

**Results:**

The results showed no significant difference among three first concentrations except for group 1 (10 µg/pulse) and group 3 (30 µg/pulse), but there was a significant difference between these groups and the fourth group (p<0.05), as this group (40 µg/pulse) statistically showed the highest rate of transfection. As the fluorescence *in situ* hybridization (FISH) results indicated the transgene was integrated in a single position in PCR positive cells.

**Conclusion:**

Increasing amount of using the vector 40µg/pulse efficiently increased the number of transfected cells. Besides of voltage and buffer strength which had been previously shown to play a critical role in electroporation efficiency, our results showed an increase in DNA concentration could affect an exponential surge in the electroporation efficiency.

## Introduction

Gene transfer can be performed using any of the biological, chemical or physical techniques which have been successfully used in the past (1). Various strategies have been used to introduce exogenous DNA into fetal fibroblasts such as lipid based delivery (2, 3), viral delivery (4, 5), and electroporation (6-8). Although these methods have successfully been used to produce transgenic animals, optimal conditions for each of these strategies which are capable of producing expected results regarding exogenous DNA delivery into the cytoplasm of fetal fibroblast have not been explained.

One of such techniques is electroporation, a physical technique, which is safe simple and inexpensive. Electroporation is a DNA transfer technique based on the application of electric pulses to briefly permeabilize the cellular membrane and to drive the negatively charged DNA along the electric field inside the cells (9). It is mostly used *in vitro* to transfect cell types which show low efficiencies of DNA uptake using other techniques, such as lipofection or calcium–phosphate transfection (10-12). Of the latter techniques, electroporation provides the most constant conditions and, as a result, it has the highest efficiency (13). Other techniques, such as lipofection, microinjection and calcium phosphate precipitation can also be used to transfer DNA into different cells; however, problems have been encountered using some of the aforementioned techniques causing low efficiency results. For example, lipofection shows low efficiency, despite the high numbers of cells remaining alive after applying the pulse. In addition, although microinjection is broadly applicable to mammalian cells and to protoplasts, it is grueling and generally inapplicable to intact microorganisms with cell walls (14, 15).

As yet, several steps have been recognized for gene electro transfer as mentioned by Anze Zupanic et al. (16), including: "electropermeabilization of the cell membrane, contact of pDNA with the cell membrane (formation of a DNA-membrane complex), and translocation of pDNA across the membrane, transfer of pDNA to and into the nucleus and gene expression" (17-20). Therefore, during the exposure to electric pulses, the plasmid DNA does not enter the cell, but is 'trapped into' the permeabilized membrane (21). A recent study by Faurie et al. (19) have showed the existence of two categories of plasmid DNA/membrane interaction; a metastable plasmid DNA/membrane complex from which plasmid DNA can leave and return to external medium, and a stable plasmid DNA/membrane complex, where plasmid DNA cannot be removed even with the help of electric pulses of reversed polarity. Only plasmid DNA belonging to the second category results in effective gene expression.

The efficacy of electroporation and gene electrotransfer depends on pulse parameters including amplitude, duration, number, pulse repetition frequency and geometric properties of the electrode and tissue/sample structure (22-25). These parameters define the duration of exposure to external electric field and the electric field strength, which have been shown to be the most important parameters in cell electroporation (16).

The fetal fibroblastsare utilized as the most used somatic cells for transgenic livestock production because they are collected and cultured easily, capable of being modified genetically and posse the ability to produce live offspring (26). Also, these cells have many features such as the reproducible ability of making cloned animals, a doubling time and life span that make them appropriate for genetic modifications by the utilization of selectable markers such as Geneticin.

This study was conducted to increase transfection efficiency of electroporation using different concentrations of DNA *in vitro* in goat fetal fibroblast cells and eventual selection of transgenic fetal fibroblasts colonies to be used for the production of genetically modified cloned goats. This method has provided effectively the ability to produce fetal fibroblast lines that are probably transgenic and capable of producing cloned offspring.

The objective of this experiment is to produce randomly integrated transgenic cell lines for generation of transgenic goat which is capable of secreting recombinant human coagulation factor IX (rhcfIX) in the milk. To perform this experiment, a gene construct was made and inserted into the expression cassette.

## Materials and Methods

### Isolation of goat fetal fibroblasts

In this experimental study, the goat fetal fibroblast cells used as karyoplast donors were prepared as previously described (27). The lines of female fibroblast cells were established from day- 35 fetuses. The fetus head and the internal organs were removed, and the remaining tissues were cut into small pieces (1-2 mm). Cells were cultured in Dulbecco’s modified eagle medium (DMEM; Invitrogen, USA) containing 15% fetal bovine serum (FBS; Hyclone, Logan, Utah, USA), 2 mM L-glutamine and 1 mM sodium pyruvate, and were seeded into 25 cm2 tissue culture flasks. After three sub passages, the cells were frozen with 10% dimethy sulfoxide (DMSO) and stored in liquid nitrogen. Before transfection, the cells were analyzed for normal chromosome count by Giemsa staining method and were sexed by cytogenetic techniques (karyotype).

### Production of gene construct

 The cDNA encoding human coagulation factor IX (hfIX) was made from liver cells by reverse transcription polymerase chain reaction (RT/PCR). The primers contained *XhoI* sites in the 5' end for the synthesis of hfIX cDNA and sub-cloning of pBC1 (Forward: 5'-CTCGAGCCACCATGCAGCGCGTGAACATGATC- 3' Reverse: 5'-CTCGAGTCATTAAGTGAGCTTTGTTTTTTCCTTA -3'). The PCR amplification consisted of 30 cycles with annealing at 58℃ for 30 seconds and extension at 72℃ for 45 seconds. The PCR product was cloned into the T vector (pTZ57R/T-Fermentas, USA) followed by digestion and sequencing. Then, the hfIX cDNA was exited from the T-vector by *XhoI*, and consequently, it was cloned into the pBC1 entailing beta-casein promoter (Invitrogen, USA). Finally, in order to integrate the gene construct into the genome, ampicillin resistance gene was removed from the construct by ligation at the SalI and NotI sites, and the linear construct was made.

### Production of gene construct

 About 10^7^ cells were harvested at 90% confluence, mixed with 10 to 40 microgram/µl linear gene-targeted vector (10^7^ cells in 425 µl of dulbecco’s phosphate buffered saline (DPBS) with different concentration of the gene), transferred into a 0.4 cm cuvette (Bio-Rad laboratories, 2000), and subjected to a pulse of 217-218 Volts delivered by a Gene Pulser (Bio-Rad München, Germany). This section repeated 3 times. Cells and DNA were incubated in DMEM medium containing 10% FBS for 15 minutes at room temperature. The transfected cells were plated into 10 cm dishes in DMEM without selection. After 24 hours, culture medium of cells was exchanged.

### Identification of transfected by PCR

Three to five days after transfection, parts of the cells were considered for PCR analysis in order to identify transfected cells. The remaining cells were expanded by passaging until sufficient cells were obtained for cryopreservation. Genomic DNA was extracted by phenol/chloroform standard protocol and used as a template for PCR using designed primer pair to enable the amplification of a fragment of the hFIX cDNA. PCR analysis was also performed on the primary cultured fibroblasts and their corresponding supernatant. The supernatant was considered in the PCR analysis to rule out the possibilities of false positive results due to floating DNA in the supernatant. The transfected cells were thawed and seeded into six-well plates at a density of 1.3×10^4^ cells per well (dilution 1:10). After six days of selection, each well was divided into two parts: the first part was used for additional passages, and cells from the second part were harvested for PCR analysis. Subsequently, positive cells were isolated for fluorescence *in situ* hybridization.

### Fluorescent in situ hybridization (FISH)

#### Probe preparation

Mini-Prep extracted recombinant Plasmid (pBC1- hFIX) was labeled with the traditional nick translation method (Vysis DNA labeling kit, Abbott Molecular, USA) and Spectrum Orange-dUTP (Vysis, Abbott Molecular, USA) according to manufacturer’s instruction.

#### Slide preparation

Metaphase slides were prepared out of mixed cultures of transfected fibroblasts and non-transfected ones according to standard cytogenetic procedures for metaphase slide preparation.

#### Hybridization

Slides burdened with cytoplasm were treated 3 minutes in 0.05% Pepsin/0.01 N HCL at 37℃. Then, they were placed in 1X phosphate buffered saline (PBS) buffer for 5 minutes at room temperature (RT) to neutralize pepsin protease activity followed by washing pepsin aggregates off the slides. Thereafter, prepared metaphase slide was soaked in 2X saline-sodium citrate (2X SSC) buffer (pH=7.0) at 37℃ for 10 minutes. Next, slides were dehydrated by soaking in 70%, 85%, and 100% ethanol at RT for 2 minutes each time, then allowed to air dry. Two µL of the probe with 8 µL of LSI/WCP hybridization solution (Vysis, Abbott Molecular, USA) were mixed and applied to each slide followed by covering with a 18X18 mm² cover slip and sealing with rubber cement. Slide containing probe, at the co-denaturation step, was baked simultaneously for 5 minutes in a 75℃ stable slide warmer. Probe/slide assembly was incubated in a 37℃ humidified chamber overnight. For post hybridization washes, slides were washed in 0.4X SSC/0.3% NP-40 at 73℃ for 2 minutes, and then transferred into 2X SSC/0.1% NP-40 at RT for 1 minute. Ten µL of DAPI/antifade (Cytocell) was applied to a slide and covered with a 20X20 mm² glass cover slip. Hybridized specimen was scored using a 100 Watt epi-fluorescence microscope (Olympus-BX51, Japan) equipped with spectrum-orange filter and 100X Objective.

#### Statistical analysis

Descriptive statistics are presented as percent and odds ratio. Logistic regression was used to evaluate the association between number of positive clones and DNA concentration. The value of p<0.05 was considered to be statistically significant. Data were analyzed using Stata version 12 (Stata Corp., College Station, TX, USA).

## Results

**Fig 1:**
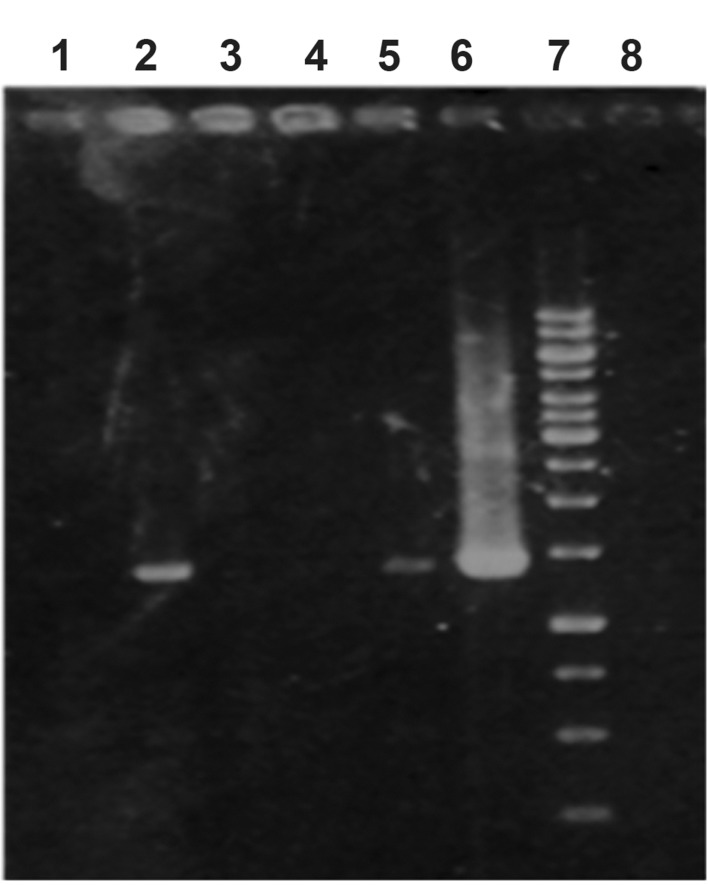
Confirmation of transfected cells by PCR. Lanes 1, 3 and 4; negative transfected cells, Lanes 2 and 5; positive transfected cells, lane 6; positive control, lane 7; 1Kb ladder and lane 8; negative control.

The conditions reported in this manuscript, specifically, from the total number of 120 cultivated wells that were analyzed by polymerase chain reaction (for each 10 µg/pulse-, 20 µg/pulse-, 30 µg/ pulse-, 40 µg/pulse-experiment), respectively, 0; 1; 2; and 5 transfected wells were found at first repeat, 1; 2; 2; and 6 transfected wells were found at second repeat, and finally 0; 0; 3; and 6 transfected wells were found at third repeat (Fig1).

**Fig 2:**
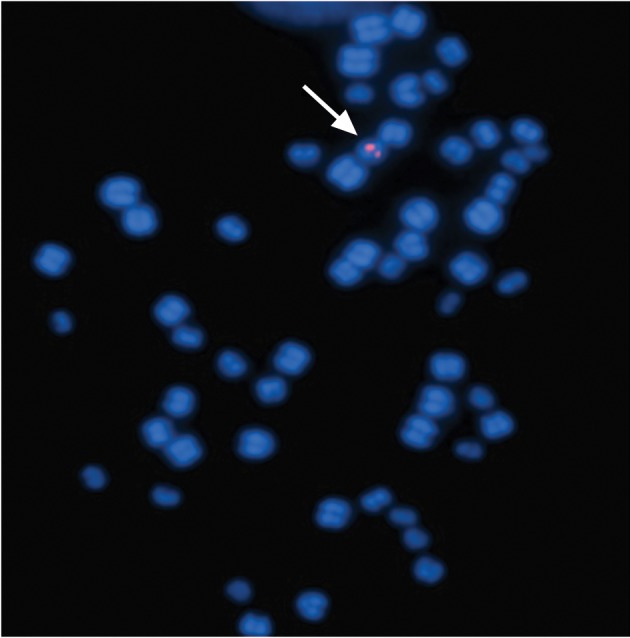
Single integration site of one of the transfected cell lines is mapped by FISH analysis.

Subsequently, the positive samples were subjected for FISH using whole gene construct as a probe. The result of fluorescence in situ hybridization also revealed that transgene integration in goat fetal fibroblasts, regardless of copy number, occurred within a single location in PCR positive cells (Fig 2).

Development of transgenic animals requires effective delivery of naked DNA into the donor cell to be used for nuclear transfer. Statistical analysis revealed significant differences between the first three concentrations and the final group of 40µg/pulse (p<0.05). No significance difference was seen between DNA concentrations of 10µg/pulse and 20µg/pulse, but there is a significant difference between concentrations of 10µg/pulse and 30µg/pulse (p<0.05). No significance difference was detected between DNA concentrations of 10µg/pulse and 20µg/ pulse, either (Table 1).

**Table 1:** Logistic regression analysis and cross table result for association between the numbers of positive clones and DNA concentrations


Variable	Number ^a^ (%)	OR ^a^	95% CI ^a^ for OR	P value

DNA concentration (µg/pulse)				
10	1 (3.3)	0.026	(0.003-0.219)	0.001
20	3 (10)	0.084	(0.021-0.342)	0.001
30	7 (23.3)	0.232	(0.076-0.707)	0.01
40	17 (56.7)	Reference group		


a; number of positive clones, CI; confidence interval and OR; odds ratio.

## Discussion

We have found that increasing of DNA concentration leads to successfully introduction of exogenous DNA into the goat fetal fibroblasts. Furthermore, the mentioned-conditions produce stably integrated transgenic cell lines in order to production of transgenic goats following nuclear transfer.

Introducing exogenous DNA into the genome of animals has almost been a simultaneous provision with recombinant DNA technology since a few years ago (28-30). A study conducted by Ross et al. (26) has showed that an approach leading to the identification of optimal electroporation conditions results successfully to introduction of exogenous DNA into the cytoplasm of porcine fetal fibroblasts.

In this study the relationship between the quantity of naked DNA and transformation efficiency was examined. Our aim was to demonstrate a simple way to enhance the efficiency of electroporation. As the results showed, the transformation rate varied depending on the DNA concentration. We observed a significant increase in transfection colonies in response to increase of DNA concentration to 40 µg/pulse.

Using a 40 µg/pulse DNA concentration, we achieved six positive clones. However, only one positive clone was gained through the lowest concentration (10 µg/pulse). As it is illustrated in table 1, the transformation rate used the 40 µg/pulse was higher than that one used the other concentrations. Our obtained result confirmed that the highest transformation rate belonged to 40 micrograms of naked DNA per pulse.

## Conclusion

All and all, the results showed increasing the DNA concentration resulted in a higher electroporation output. Therefore, it can be concluded that a 6-fold linearized naked DNA absorption was performed by incremental concentrations. However, it is possible that this finding may not be relevant to all the different types of DNA, in particular to plasmids. But, the effect of increasing DNA concentration on the rate of electroporation is evident. This condition has resulted in the production of stable and viable transgenic cell lines. Therefore, irrespective of other critical factors such as cell type and buffer composition, during electroporation of different concentration of DNA can directly affect the result of transfection. Present study indicated that the utilization of linearized naked DNA is a credible method by which to identify the ability of electroporation condition.
